# Genome-wide association study identified ATP6V1H locus influencing cerebrospinal fluid BACE activity

**DOI:** 10.1186/s12881-018-0603-z

**Published:** 2018-05-11

**Authors:** Hao Hu, Haiyan Li, Jieqiong Li, Jintai Yu, Lan Tan, Alzheimer’s Disease Neuroimaging Initiative

**Affiliations:** 1 Department of Neurology, Qingdao Municipal Hospital, School of Medicine, Qingdao University, No.5 Donghai Middle Road, Qingdao, 266071 Shandong Province China; 2 Department of Neurology, Weihai Wei People’s Hospital, Weihai, China; 3 Clinical Research Center, Qingdao Municipal Hospital, Qingdao University, Qingdao, China; 4 Department of Neurology, University of California, San Francisco, 675 Nelson Rising Lane, Suite 190, Box 1207, San Francisco, CA 94158 USA

**Keywords:** ADNI, Alzheimer’s disease, BACE, ATP6V1H

## Abstract

**Background:**

The activity of cerebrospinal fluid (CSF) β-site APP cleaving enzyme (BACE) is a potential diagnostic biomarker for Alzheimer disease (AD).

**Methods:**

A total of 340 non-Hispanic Caucasian participants from the Alzheimer’s Disease Neuroimaging Initiative cohort (ADNI) database were included in this study with quality-controlled CSF BACE and genotype data. Association of CSF BACE with the genetic variants of single nucleotide polymorphisms (SNPs) was assessed using PLINK under the additive genetic model. The *P* values of all SNPs for CSF BACE were adjusted for multiple comparisons.

**Results:**

One SNP (rs1481950) in the *ATP6V1H* gene reached genome-wide significance for associations with CSF BACE (*P* = 4.88 × 10^− 9^). The minor allele (G) of rs1481950 was associated with higher CSF BACE activity. Although seven SNPs in *SNX31, RORA, CDH23, RGS20, LRRC4C, MAPK6PS1* and *LOC105378355* did not reach genome-wide significance (*P* < 10^− 8^), they were identified as suggestive loci (*P* < 10^− 5^).

**Conclusion:**

This study identified rs1481950 within *ATP6V1H* influencing human CSF BACE activity, which indicated that *ATP6V1H* gene may play some roles in the pathogenesis of neurodegenerative diseases such as AD.

**Electronic supplementary material:**

The online version of this article (10.1186/s12881-018-0603-z) contains supplementary material, which is available to authorized users.

## Background

Amyloid plaque which is composed primarily of amyloid-beta (Aβ) has been reported as an important pathological change of some neurodegenerative diseases including Alzheimer’s disease (AD) [1]. Aβ is generated from the transmembrane polypeptide called amyloid precursor protein (APP) by β- and γ-secretase enzymes [1, 2]. β-secretase or β-site APP cleaving enzyme (BACE) is the important rate-limiting enzyme and its increased activity may lead to the elevation of Aβ in the brain [1, 2]. Previous studies discovered that AD subjects have increased cerebrospinal fluid (CSF) BACE enzymatic activity compared with the controls [3–12]. So researchers hypothesized that BACE enzymatic activities in CSF may become potential biomarkers for AD.

The recent development of a sensitive assay for BACE in CSF makes it feasible to study the association between CSF BACE enzymatic activity and AD [13]. Moreover, the use of quantitative traits in genome-wide association study (GWAS) has been shown to increase statistical power over case-control designs [14]. In this study we regard CSF BACE enzymatic activity as an endophenotype for a separate GWAS in the ADNI (Alzheimer’s Disease Neuroimaging Initiative database, adni.loni.usc.edu) cohort in order to discover genetic factors involved in BACE protein.

## Methods

### Alzheimer’s Disease Neuroimaging Initiative

Data used in this study were obtained from the Alzheimer’s Disease Neuroimaging Initiative (ADNI) database (www.loni.ucla.edu/ADNI). The ADNI was launched in 2003 as a public-private partnership, led by Principal Investigator Michael W. Weiner, MD, VA Medical Center and University of California–San Francisco. ADNI includes more than 800 participants ranging in age from 55 to 90. All these individuals were recruited from over 50 sites across the United States and Canada, including approximately 200 healthy controls (HC), 400 patients diagnosed with mild cognitive impairment (MCI) and 200 patients diagnosed with early AD. The AD patients were followed for 2 years and others were followed for 3 years. Structural 1.5-T magnetic resonance imaging (MRI) collected the full sample. PIB and FDG positron emission tomography (PET) imaging of a subset, some other biological markers, and performance on neuropsychological or clinical assessments were collected at baseline and at follow-up visits in 6- to 12-month intervals. The CSF BACE and genome-wide genotyping in this study were respectively available on approximately half of the cohort and the full ADNI sample. Further information about ADNI can be found in previous publications and at www.adni-info.org [15].

### Standard protocol approvals, registrations, and patient consents

This study was approved by institutional review boards of all participating institutions and written informed consent was obtained from all participants or authorized representatives.

### Participants

Our ADNI cohort included all healthy controls (HC), MCI group and AD group participants with available baseline CSF BACE samples and genotype data. To reduce the likelihood of population stratification effects in the GWAS, all the participants were restricted to non-Hispanic Caucasians. We also tested a multidimensional scaling (MDS) plot and found some genetic outliers (Additional file 1). After quality control (QC) of the CSF BACE data and removal of the outliers, there were 340 participants (AD = 86, MCI = 163, HC = 91) with CSF BACE data left. Detailed QC steps for CSF and genotype data have been previously reported and are briefly described below.

### CSF BACE measurement and quality control

Samples were obtained from 382 ADNI subjects, enrolled at 56 participating centers using previously reported methods for CSF measurements [13]. For most samples, the time from collection to freezing was within 60 min. Samples were processed, aliquoted, and stored at − 80 °C according to the ADNI Biomarker Core Laboratory Standard Operating Procedures [16].

CSF BACE proteins of all the samples were tested by a solution-based BACE enzymatic assay which has been regarded as best assay format for BACE enzymatic activity in previous studies [13]. This assay format uses a biotin labeled 15 amino acid peptide biotin-KTEEISEVNFEVEFR (NFEV) as the BACE substrate and uses a baculovirus expressed c-terminally truncated BACE (bBACE) as the BACE enzyme standard. A source of BACE using either purified recombinant truncated BACE, human or rhesus monkey CSF was co-incubated with this BACE substrate. The BACE cleavage product was then detected using an “anti-NF” neo-epitope specific rabbit polyclonal antibody and an indirect anti-rabbit horseradish peroxidase (HRP) or alkaline phosphatase (AP) development of the reaction [13]. The BACE activity assay includes two steps (1 Enzyme+substrate, 2 ELISA to measure product) [13]. Luminescence from assay plates was read on EnVision (PerkinElmer, model 2104). The counts from individual CSF samples were converted to BACE enzymatic activity using coefficients determined by a quadratic fit to the bBACE standard curve [13].

Mean and standard deviations (SD) baseline of CSF BACE measures were calculated by observers blind to diagnostic information and subjects who had a value greater or smaller than 3-fold SD from the mean value were regarded as extreme outliers and removed from the analysis.

### Genotyping and quality control

Single nucleotide polymorphism (SNP) genotyping for more than 620,000 target SNPs was completed on all ADNI participants using the following protocol. A total of 7 mL blood of each participant was taken in EDTA containing Vacutainer tubes and genomic DNA was extracted using the QIAamp DNA Blood Maxi Kit (Qiagen, Inc., Valencia, CA) following the manufacturer’s protocol. EBV-transformed B lymphoblastoid cell lines were established. Genomic DNA samples were analyzed using the Human 610-Quad BeadChip (Illumina, Inc., San Diego, CA) according to the manufacturer’s protocols (Infinium HD Assay; Super Protocol Guide; rev. A, May 2008).

Stringent QC assessment was performed using the PLINK software package (http://pngu.mgh.harvard.edu/purcell/plink/), release v 1.07,15 as described previously. Stringent QC assessment followed these criteria: minimum call rate for SNPs and individuals > 95%, minimum minor allele frequencies (MAF) > 0.05, Hardy-Weinberg equilibrium test *P* > 0.001. The restriction to SNPs with a MAF greater than 5% served to reduce the likelihood of false-positive results in the context of modest sample size to enhance statistical power [14]. What’s more, elimination of relatively rare markers reduced the severity of the multiple comparison correction which in turn enhanced statistical power. After the QC procedure, all 340 participants remained in the analysis and only 519,442 out of 620,901 SNPs remained in the analysis. The overall genotyping rate for the remaining dataset was 99.5%.

### Statistical analyses

To find the association of CSF BACE with the genetic polymorphism, a separate GWAS was performed using PLINK software under an additive genetic model. The thresholds of *P* < 10^− 5^ and *P* < 10^− 8^ were used for suggestive and genome-wide significant associations respectively [17]. The analysis included a total of 519,442 genotyped variants. Age, gender and APOEε4 status were included as covariates. One-way analysis of variance (ANOVA) and Tukey’s multiple comparisons test were used to determine the difference of CSF BACE enzymatic activity in different diagnostic groups. The effects of genotypes on CSF BACE were examined with a multiple linear regression model. The relations between top SNP and AD-related phenotypes were also performed using PLINK software. Genome-wide associations were visualized by a software program (R, version 3.4.0; The R Foundation). Regional associations were visualized with the LocusZoom web tool (http://locuszoom.org/).

## Results

### Demographic, clinical, and cognitive characteristics

The demographic information on the final set of 340 non-Hispanic Caucasian participants and summary statistics on the CSF BACE are showed in Table 1. This study includes 86 AD subjects, 163 MCI subjects and 91 HC subjects. As shown in the Additional file 2, there was no significant difference in gender (AD *P* = 0.32470, MCI *P* = 0.26220, HC *P* = 0.82720, Total *P* = 0.81770) among the three diagnostic groups. For age (Additional file 3), though we did not find the linear correlation in the AD, MCI and total samples (AD *P* = 0.9007, MCI *P* = 0.4659, Total *P* = 0.1447), the results showed a linear correlation between age and CSF BACE activity in HC groups (*P* = 0.0063). As expected, the ε4 allele of APOE gene substantially increased the risk of AD with dose effects. Furthermore, CSF BACE enzymatic activity did not have statistical difference (*P* = 0.2762, after ANOVA and Tukey’s multiple comparisons test) among three diagnostic groups.

**Table 1:** Demographic information

Baseline diagnosis	AD	MCI	HC	Total
n	86	163	91	340
Age (years), mean ± SD (range)	75.1 ± 7.8 (56.4–89.1)	74.2 ± 7.7 (54.4–88.8)	75.5 ± 5.2 (62.0–89.6)	74.8 ± 7.1 (54.4–89.6)
Gender, male/female	47/39	109/54	49/42	205/135
*APOE* ε4 carrier (%)	45.3	33.1	13.2	30.9
CSF BACE (pM), mean ± SD	44.5 ± 17.5	48.8 ± 20.1	46.1 ± 18.0	47.0 ± 18.9

*AD* Alzheimer’s disease, *MCI* mild cognitive impairment, *HC* healthy control, *SD* standard deviation

### Characteristics of SNPs associated with CSF BACE activity

**Fig. 1:**
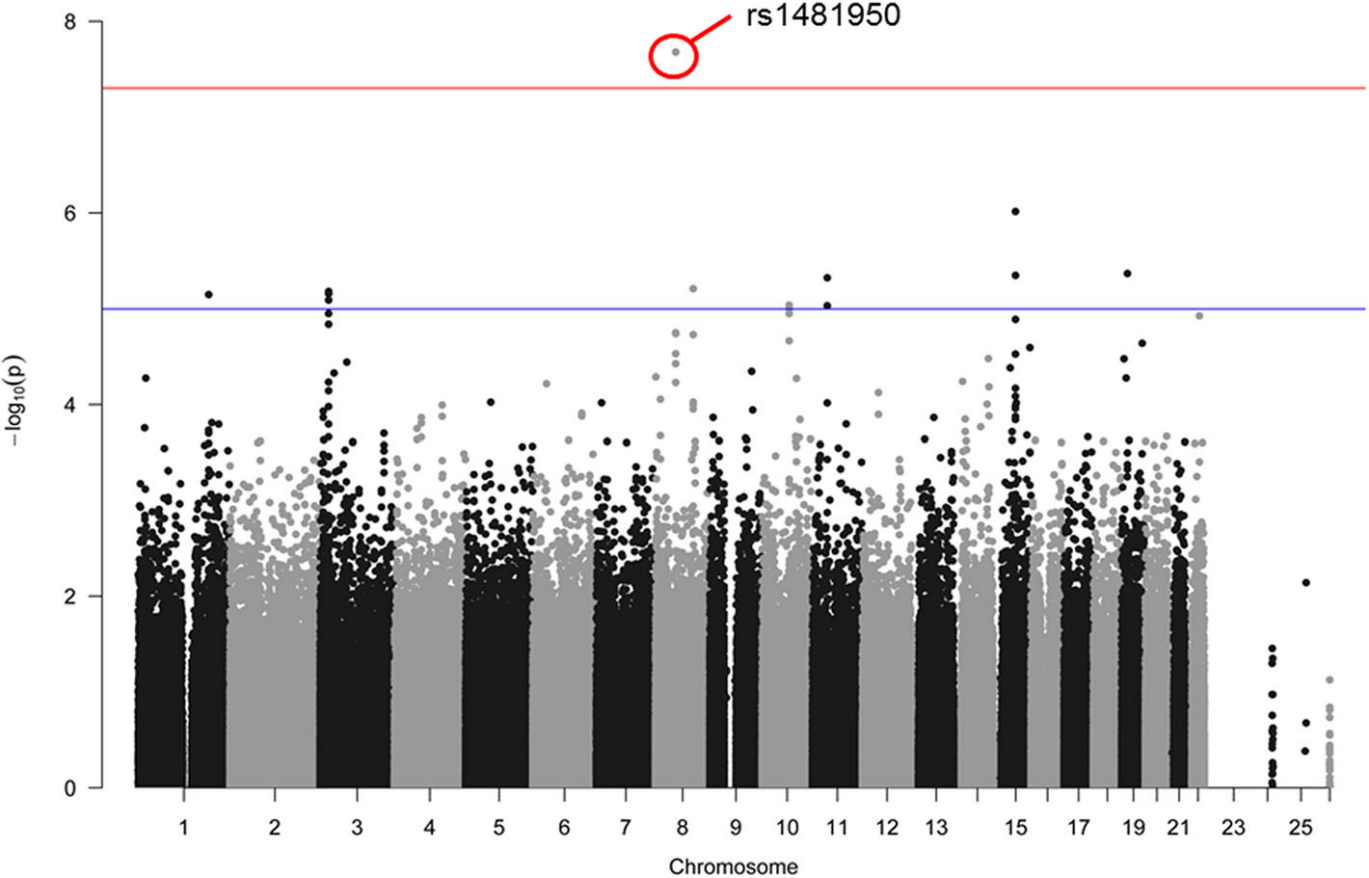
Manhattan plots for associations with CSF BACE. In the Manhattan plot, the blue and red lines represent the -log_10_ (10^− 5^) and -log_10_ (3.10 × 10^− 8^) threshold levels

**Fig. 2:**
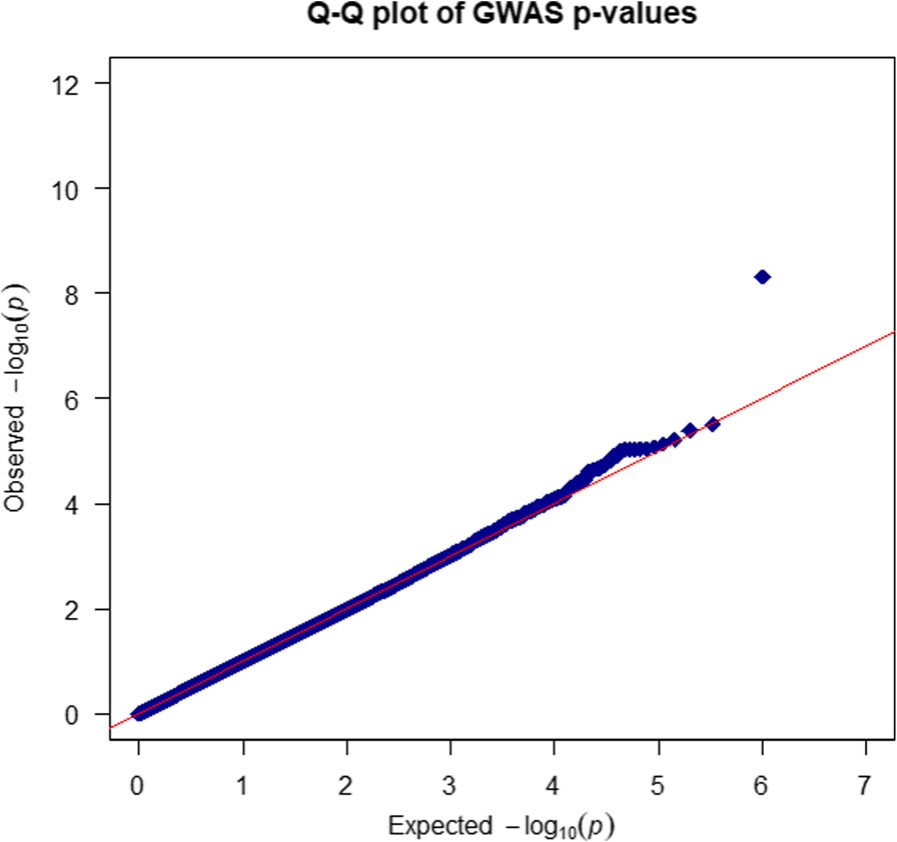
Quantile-Quantile plot

**Fig. 3:**
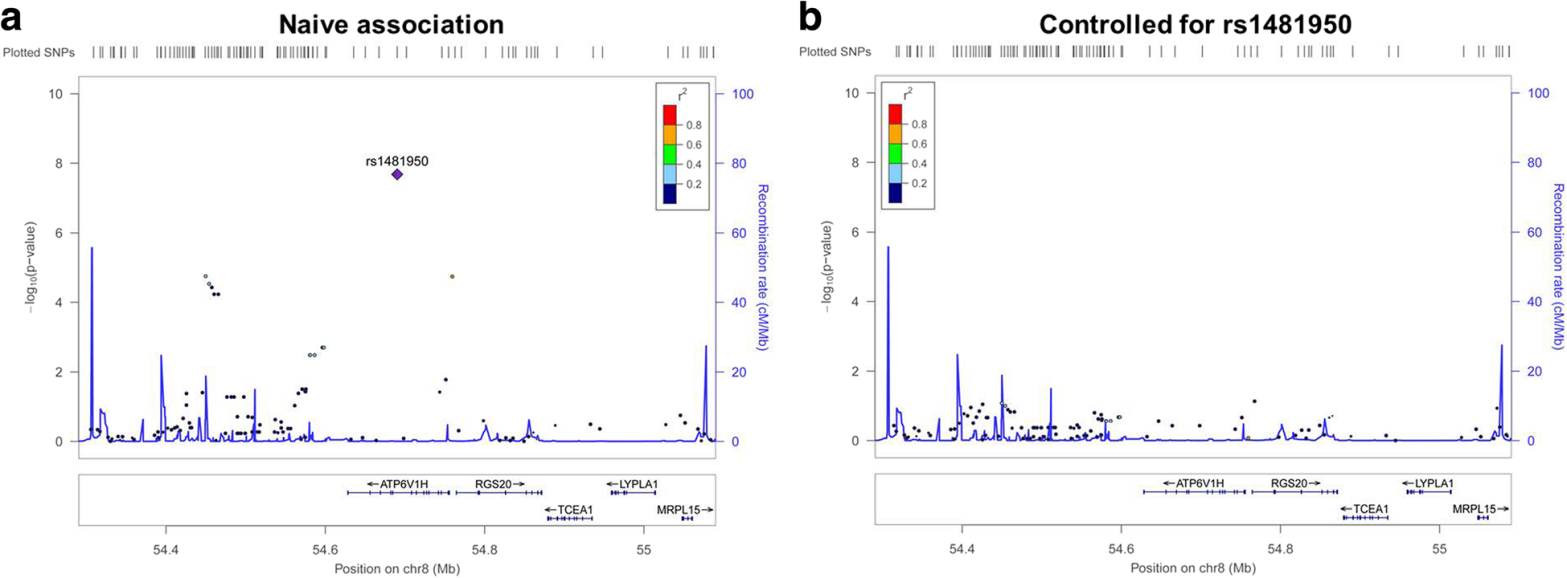
Regional plots for associations with CSF BACE. **a** Regional association results for the *ATP6V1H* region of chromosome 8. **b** Association results for chromosome 8:54290285–55090285 controlling for rs1481950

After adjusting for age, gender and APOEε4 status, one SNP (rs1481950, NM_015941.3:c.871-5558G > T) was identified associated with CSF BACE enzymatic activity at genome-wide significance levels of *P* < 10^− 8^. As shown in the Fig. 1, one SNP (rs1481950) in the region of *ATP6V1H* gene reached genome-wide significance (*P* = 4.88 × 10^− 9^). Moreover, all SNPs whose *P* values reached the level of *P* < 10^− 5^ for CSF BACE enzymatic activity and their annotation information are listed in Table 2. Although these SNPs did not reach the genome-wide significance level in this limited sample, they may still have the potential significance in other larger samples. So we regarded these SNPs whose P values reached the level of *P* < 10^− 5^ as suggestive SNPs. Seven independent potential candidates (uncorrected *P* < 10^− 5^) were included (rs2507780, rs1866007, rs7903757, rs6473895, rs4075903, rs9693226, rs1900511). The Quantile-Quantile plot (QQ plot) is shown in Fig. 2 and the inflation factor is 1.The linkage disequilibrium (LD) pattern between rs1481950 and nearby SNPs is shown in Fig. 3a. These nearby SNPs showed association with CSF BACE levels with the *P* < 0.01. As the Fig. 3b shows, when the top SNP (rs1481950) was controlled, these nearby SNPs also disappeared, which indicated that these nearby SNPs were driven by the top SNP.

**Table 2:** Suggestive SNPs

SNP	Chr	Position	SNP Type	Closest RefSeq gene	Gene name	*P* value
rs2507780	8	100588738	intron	SNX31	sorting nexin 31	3.15 × 10^−6^
rs1866007	15	60526051	intron	RORA-AS1 RORA	RAR related orphan receptor A	6.02 × 10^−6^
rs7903757	10	71552620	intron	CDH23 LOC105378355	cadherin related 23	9.21 × 10^−6^
rs6473895	8	53850344	upstream variant 2 KB	RGS20	regulator of G-protein signaling 20	8.42 × 10^−6^
rs4075903	11	40186373	intron	LRRC4C	leucine rich repeat containing 4C	9.34 × 10^−6^
rs9693226	8	53540570		MAPK6PS1	mitogen-activated protein kinase 6 pseudogene 1	7.38 × 10^−6^
rs1900511	10	71559814	intron	LOC105378355		9.32 × 10^−6^

*SNP* single nucleotide polymorphism, *Chr* chromosome

### CSF BACE activity and top SNP

**Fig. 4:**
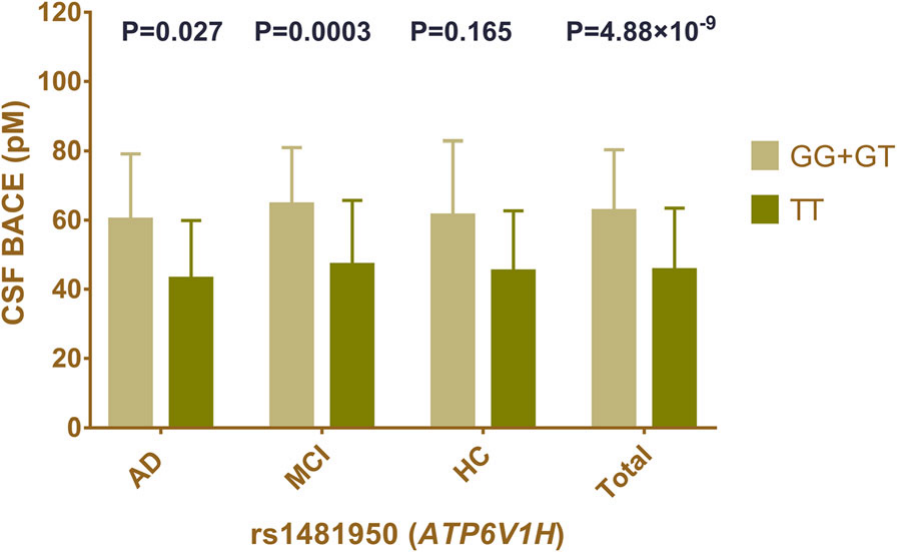
Mean CSF BACE activity as a function of baseline diagnosis and genotype. Mean and standard errors of CSF BACE activity are shown for groups defined by baseline diagnosis. The CSF BACE activity in the total samples showed positive linear association with the G allele in a multiple linear regression model after adjusting for age, gender, and APOEε4 status (GG+ GT: 63.86 ± 22.64pM, TT: 45.49 ± 17.99pM, *P* = 4.88 × 10^− 9^). The *P* value of a multiple linear regression model in each diagnosis group was displayed in the fig. (AD: *P* = 0.027 MCI: *P* = 0.0003 HC: *P* = 0.165)

All these eight SNPs were analyzed further to examine possible interactions among baseline diagnosis and genotypes on associated CSF BACE enzymatic activity by a multiple linear regression test considering age, gender and APOEε4 status as covariates. For the top SNP (rs1481950), as indicated in Fig. 4, CSF BACE activity in the whole samples showed positive linear association with the G allele in a multiple linear regression model after adjusting for age, gender, and APOEε4 status (GG + GT: 63.86 ± 22.64pM, TT: 45.49 ± 17.99pM, *P* = 4.88 × 10^− 9^, Beta: 22.48, 95% CI: 15.22 ~ 29.75). Further analysis found this linear association between genotype and CSF BACE activity in both AD (GG + GT: 60.14 ± 19.13pM, TT: 43 ± 16.85pM, *P* = 0.02720 Beta: 0.23820, 95% CI: 0.02137 ~ 0.43360) and MCI groups (GG + GT: 69.36 ± 23.99pM, TT: 46.99 ± 18.72pM, *P* = 0.00030, Beta: 0.28280, 95% CI: 0.13040 ~ 0.42210). However, in HC group, results did not show significant differences in CSF BACE activity among three genotype subgroups in both ANOVA (*P* = 0.06370) and multiple linear regression analysis (*P* = 0.16480, Beta: 0.14690, 95%CI: -0.06708~ 0.34790).

### Top SNP and AD-related phenotypes

**Fig. 5:**
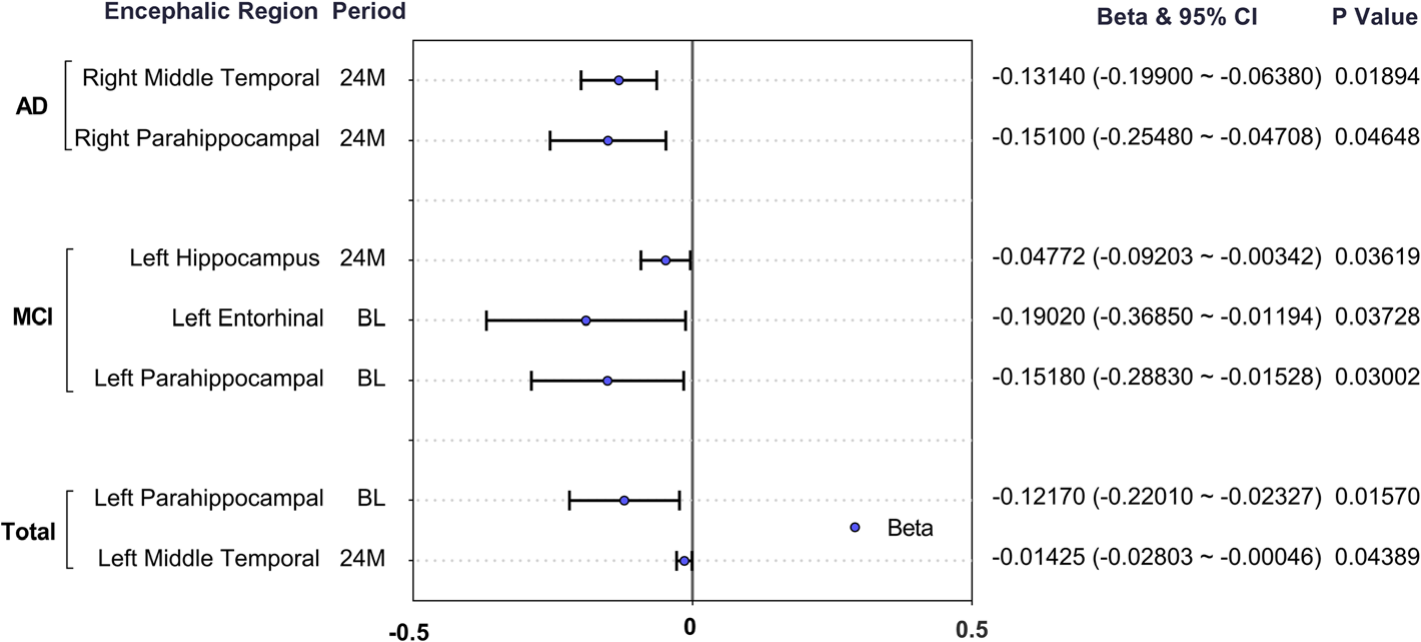
Forest plot of rs1481950 and AD-related encephalic regions in ADNI. 24 M: The follow-up period is 24 months; BL: Baseline; CI: credibility interval

This study also tested relations between rs1481950 and AD-related phenotypes. In all samples, results showed that mutations of rs1481950 had significant correlations with the atrophy of middle temporal gyrus (Beta: -0.01425, 95% CI: -0.02803 ~ − 0.00046, *P* = 0.04389) and parahippocampal gyrus (Beta: -0.12170, 95% CI: -0.22010 ~ − 0.02327, *P* = 0.01570). Similarly to this result, in AD patients, results also showed that mutations of rs1481950 had significant correlations with the atrophy of middle temporal gyrus (Beta: -0.13140, 95% CI: -0.19900 ~ − 0.06380, *P* = 0.01894) and parahippocampal gyrus (Beta: -0.15100, 95% CI: -0.25480 ~ − 0.04708, *P* = 0.04648). In MCI patients, results showed that mutations of rs1481950 had correlations with the atrophy of hippocampus (Beta: -0.04772, 95% CI: -0.09203 ~ − 0.00342, *P* = 0.03619), entorhinal cortex (Beta:-0.19020, 95% CI: -0.36850 ~ − 0.01194, *P* = 0.03728) and parahippocampal gyrus(Beta: -0.15180, 95% CI: -0.28830 ~ − 0.01528, *P* = 0.03002) (Fig. 5). For HC groups, though we did not find some correlations between rs1481950 and brain atrophy, the results showed that mutations of rs1481950 had significant correlations with the CSF phosphorylated tau protein (p-tau) (Beta: 8.52600, 95% CI: 2.74700 ~ 14.31000, *P* = 0.00426).

## Discussion

In this multicenter study, we identified a genome-wide significant association of a SNP (rs1481950) in the gene *ATP6V1H* region with CSF BACE activity and found seven additional suggestive association loci. Interestingly, this study is the first to show that the rs1481950 risk variant in *ATP6V1H* significantly affects CSF BACE activity. We found statistically significant differences in CSF BACE activity between the two genotype groups (GG + GT and TT). It is worth noting that this statistical differences between gene polymorphism of *ATP6V1H* and CSF BACE activity existed in both AD (*P* = 0.0027) and MCI groups (*P* = 0.0003). These results indicated that the rs1481950 risk variant (G) in *ATP6V1H* might significantly increase the CSF BACE activity especially in the AD and MCI individuals.

Rs1481950 is in an intronic region. Current studies haven’t found the exact mechanisms of impairing the expression of *ATP6V1H*. However, the roles of intronic regions which have been ignored all the time have attracted the attention of scientists. And more and more studies have proved that intronic region may play some important roles in controling the initiation and termination of transcription. Moreover, common molecular mechanisms for an intronic SNP to alter mRNA levels are to affect transcription, RNA elongation, splicing, or maturation [18–22].

The ATPase, H^+^ transporting, lysosomal 50/57 kDa, V1 subunit H gene (*ATP6V1H*) at Chr8q11.2 encodes for the V1H subunit of vacuolar ATPase (V-ATPase) [23, 24]. *ATP6V1H* gene was mainly studied and discussed about its roles in diabetes in previous researches and the data showed that the down-regulation of its gene expression correlates with the presence of type-2 diabetes [25]. Though there is still no research point out a direct relation between *ATP6V1H* gene and AD, some studies about encoded protein and metabolic process of BACE indicate that mutations of *ATP6V1H* gene may contribute to the increased BACE activity. V-ATPase belongs to the rotary ATPase family and is a multiprotein membrane complex. The most important function of V-ATPase is to acidify intracellular compartments by using the energy gathered from ATP hydrolysis to pump protons [23]. One of the most important influence factors of BACE activity is PH. Acidic Intracellular environment (PH = 4.5) is optimal for BACE activity. So the dysfunction of V-ATPase may lead to the change of the acidic Intracellular environment and then influence the BACE activity. BACE activity is also associated with mature processing. The mature process of BACE requires the formation of disulfide bonds, glycosylation and some other modification processes [26]. These steps occurs in the endoplasmic reticulum and golgi body and may be influenced by the changes of the intracellular environment. Moreover, V-ATPase also plays an important role in lysosomal acidification. Lysosomal pathway is an important degradation pathway of BACE protein [27, 28]. To sum up, V-ATPase may play some important roles in both BACE protein levels and activity.

The results also showed that mutations of rs1481950 had significant correlations with the atrophy of AD-related encephalic regions including middle temporal gyrus, parahippocampal gyrus, hippocampus and entorhinal cortex. Moreover, the results showed that mutations of rs1481950 had significant correlations with the CSF p-tau. These results indicated that mutations of rs1481950 may relate to AD and may influence the volume of AD-related encephalic regions by changing the metabolism of some AD-related proteins such as tau.

In this study, we did not find any significant differences in CSF BACE activity among the three diagnostic groups. This aspect of our research is in line with two previous studies including an ADNI cohort study [29, 30]. Some previous studies show different results including increased activity in MCI and AD [31], and increased BACE1 activity in MCI but not in AD [13, 32]. These inconsistent results may be explained by the characteristics of the study samples, the wide range of BACE1 activity measurements, and the large overlap between the groups.

There are several potential limitations of this study. First, the ADNI-1 sample was limited in sample size when CSF BACE data, different genotypes and diagnosis subgroups were taken into consideration, which makes effective sample size small for some tests. Thus, it will still be necessary to replicate these findings in a larger dataset. Second, recent studies also indicated that except CSF BACE, plasmatic BACE was also associated with AD [33]. The associations among gene polymorphism, CSF BACE, plasmatic BACE and AD still need further study. Third, our sample was restricted to Caucasians to avoid genetics stratification across ethnicities, while all the genes may show different frequencies and polymorphisms in different populations. The relationships between these genes and AD need to be tested in more populations. Fourth, the ADNI database did not cover the detailed classification of the AD patients (sporadic and familial; early onset and late onset). So further studies with a better profile of AD patients should be tested for comparative purposes with other studies.

## Conclusions

In summary, after a separate GWAS of CSF BACE, we found a top SNP (rs1481950) in *ATP6V1H* gene with the *P* value reaching genome-wide significance and seven suggestive SNPs with the P value lower than 10^− 5^. Rs1481950 risk variant (G) in *ATP6V1H* may increase the CSF BACE activity. Seven genes (*SNX31, RORA, CDH23, RGS20, LRRC4C, MAPK6PS1, LOC105378355*) were regarded as candidate genes. These results provide clues to some novel pathogenic genes associated with some BACE related diseases, such as AD. The in-depth discussion and study of these associations can help us to find the exact mechanisms of AD, which may indicate some new diagnostic methods and therapeutic directions.

## Additional files

Additional file 1: The MDS plot of samples. (TIF 8989 kb)

Additional file 2: The correlation between gender and CSF BACE activity. (TIF 2480 kb)

Additional file 3: The correlation between age and CSF BACE activity. (TIF 2436 kb)

## Abbreviations

AD: Alzheimer disease
ADNI: Alzheimer’s Disease Neuroimaging Initiative
ANOVA: One-way analysis of variance
AP: Alkaline phosphatase
APP: Amyloid precursor protein
Aβ: Amyloid-beta
BACE: β-site APP cleaving enzyme
bBACE: Baculovirus expressed cterminally truncated BACE
CSF: Cerebrospinal fluid
GWAS: Genome-wide association study
HC: Healthy controls
HRP: Horseradish peroxidase
LD: Linkage disequilibrium
MAF: Minor allele frequencies
MCI: Mild cognitive impairment
MRI: Magnetic resonance imaging
PET: Positron emission tomography
QC: Quality control
SD: Standard deviations
SNPs: Single nucleotide polymorphisms
