# Supplementary figures and images for: Genome-wide association study identified ATP6V1H locus influencing cerebrospinal fluid BACE activity
Source: BMC Med Genet. 2018 May 11;19:75. doi: 10.1186/s12881-018-0603-z (PMC5948839; doi:10.1186/s12881-018-0603-z)

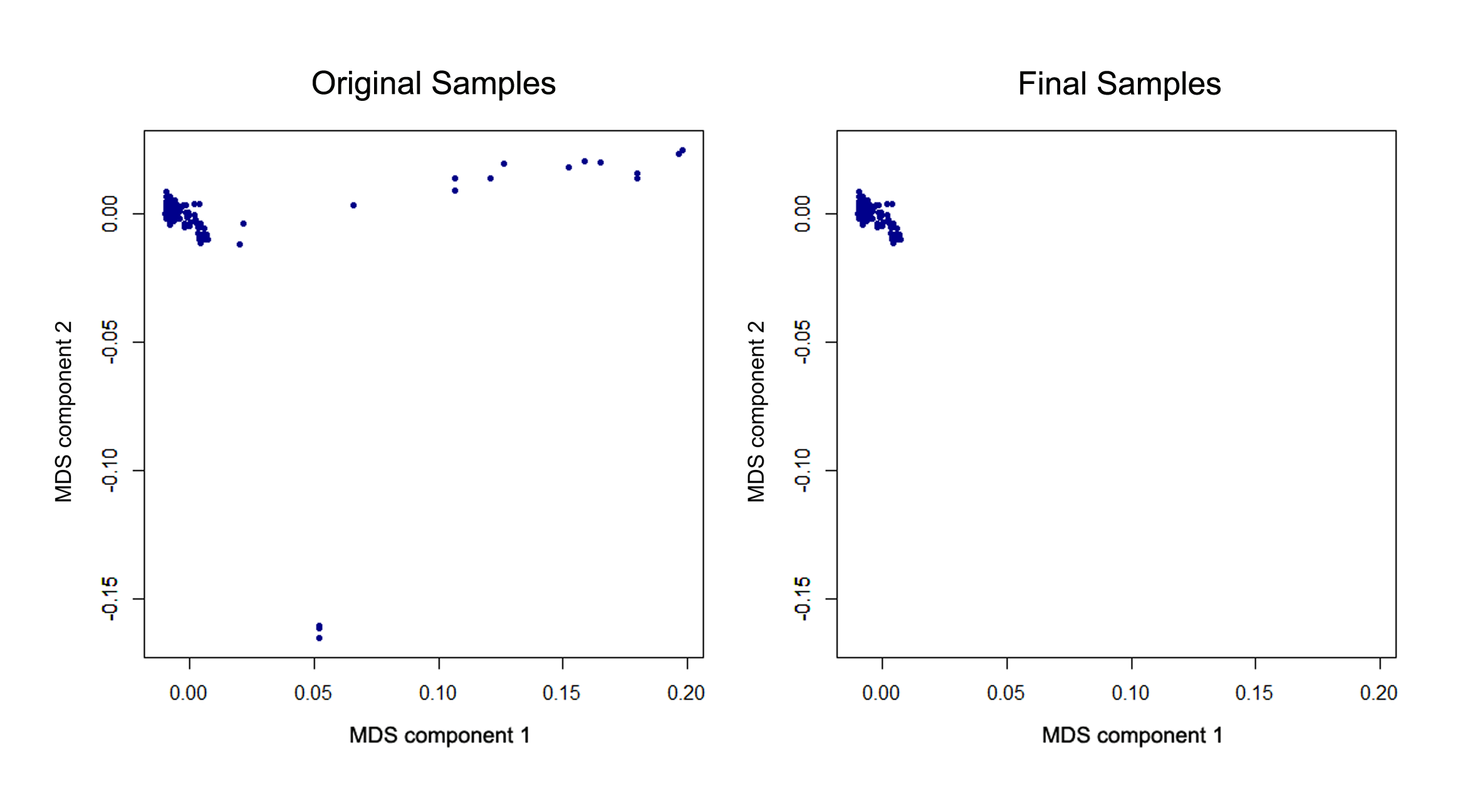

Supplement: Supplementary file 1 — The MDS plot of samples. (TIF 8989 kb) [file 12881_2018_603_MOESM1_ESM.tif]

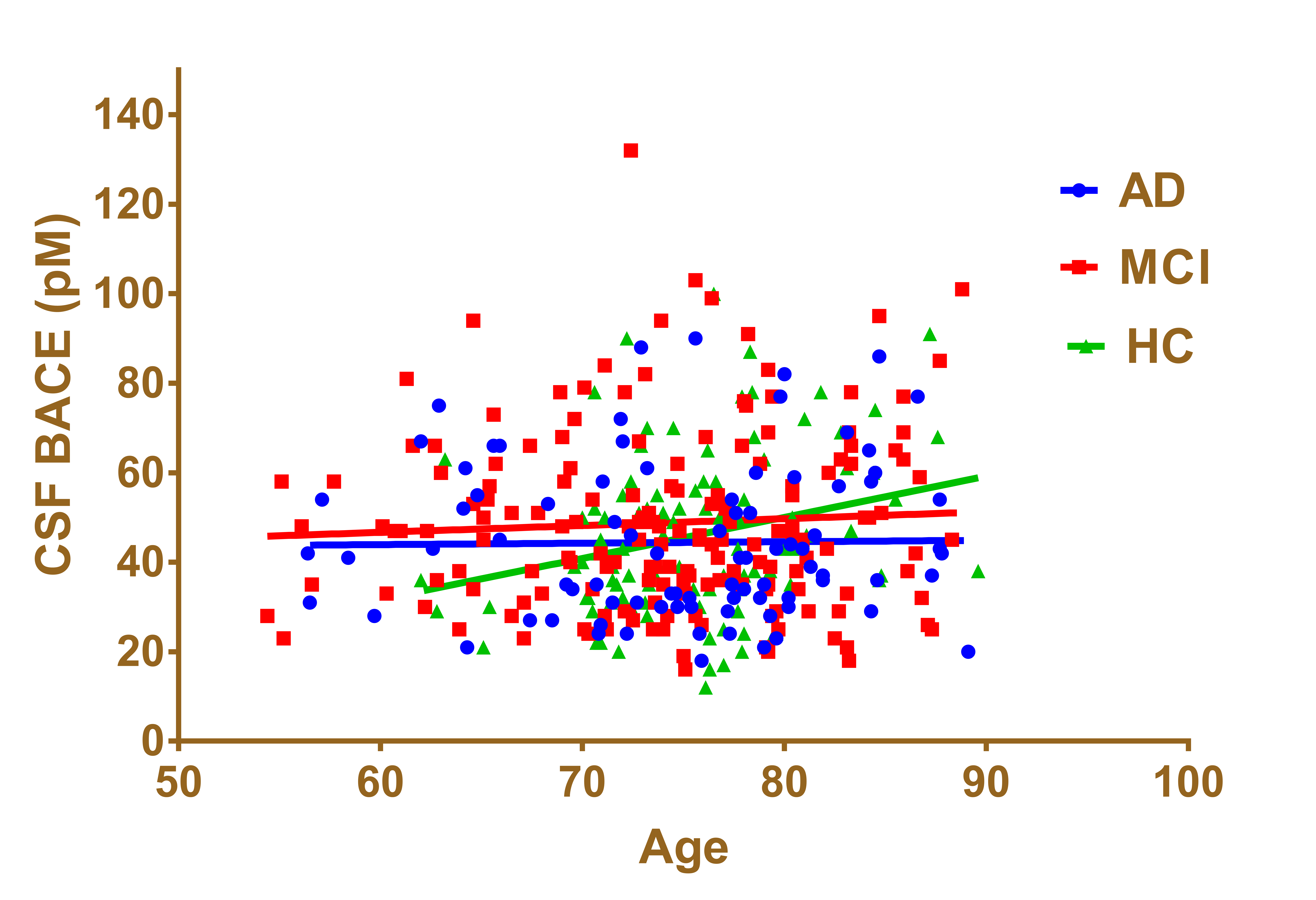

Supplement: Supplementary file 3 — The correlation between age and CSF BACE activity. (TIF 2436 kb) [file 12881_2018_603_MOESM3_ESM.tif]
